# Identification of suicidal behavior among psychiatrically hospitalized adolescents using natural language processing and machine learning of electronic health records

**DOI:** 10.1371/journal.pone.0211116

**Published:** 2019-02-19

**Authors:** Nicholas J. Carson, Brian Mullin, Maria Jose Sanchez, Frederick Lu, Kelly Yang, Michelle Menezes, Benjamin Lê Cook

**Affiliations:** 1 Health Equity Research Lab, Cambridge Health Alliance, Cambridge, MA, United States of America; 2 Department of Psychiatry, Harvard Medical School, Boston, MA, United States of America; 3 Prevention and Community Health Department, Milken School of Public Health, George Washington University, Washington, D.C., United States of America; 4 Department of Psychiatry, Albert Einstein College of Medicine, Bronx, NY, United States of America; 5 University of Virginia, Charlottesville, VA, United States of America; National Institutes of Health, UNITED STATES

## Abstract

**Objective:**

The rapid proliferation of machine learning research using electronic health records to classify healthcare outcomes offers an opportunity to address the pressing public health problem of adolescent suicidal behavior. We describe the development and evaluation of a machine learning algorithm using natural language processing of electronic health records to identify suicidal behavior among psychiatrically hospitalized adolescents.

**Methods:**

Adolescents hospitalized on a psychiatric inpatient unit in a community health system in the northeastern United States were surveyed for history of suicide attempt in the past 12 months. A total of 73 respondents had electronic health records available prior to the index psychiatric admission. Unstructured clinical notes were downloaded from the year preceding the index inpatient admission. Natural language processing identified phrases from the notes associated with the suicide attempt outcome. We enriched this group of phrases with a clinically focused list of terms representing known risk and protective factors for suicide attempt in adolescents. We then applied the random forest machine learning algorithm to develop a classification model. The model performance was evaluated using sensitivity, specificity, positive predictive value (PPV), negative predictive value (NPV), and accuracy.

**Results:**

The final model had a sensitivity of 0.83, specificity of 0.22, AUC of 0.68, a PPV of 0.42, NPV of 0.67, and an accuracy of 0.47. The terms mostly highly associated with suicide attempt clustered around terms related to suicide, family members, psychiatric disorders, and psychotropic medications.

**Conclusion:**

This analysis demonstrates modest success of a natural language processing and machine learning approach to identifying suicide attempt among a small sample of hospitalized adolescents in a psychiatric setting.

## Introduction

In 2017, 17.2% of U.S. high school students reported having seriously considered attempting suicide and 7.4% reported having attempted suicide in the past year [1]. Rates of completed suicide among adolescents continue to increase [2]. Lifetime risk factors for suicide are well-established for adolescents who receive inpatient psychiatric treatment [3–5]. Prior suicide attempt is an especially potent risk factor for future suicide attempts [6], but some adolescents may not be willing to share such history with clinicians or family during the inpatient stay [7–9]. This reluctance to disclose might be due to the repercussions of disclosing to clinicians or family, including feelings of shame, loss of privacy or privileges, mistrust of healthcare providers, fears of further restrictions (e.g. a longer hospital stay), or under-estimating the severity of past behaviors [10]. In addition, adolescents hospitalized after acute suicidal behavior remain at high risk in the initial months following discharge [5, 11–14].

Existing clinical tools for assessment of suicide risk or prior attempt can be time-intensive, costly, and might require clinician administration [15–17]. Therefore, a computerized algorithm developed from clinical notes and integrated into a hospital’s electronic health record could function as an innovative and efficient complement to the judgment of the clinical team to classify whether a hospitalized patient, often whom the clinical team is meeting for the first time, has a history of suicide attempt. The National Action Alliance for Suicide Prevention’s Research Prioritization Task Force has identified the development of systems using healthcare data as a promising approach to the “retrospective examination of pathways leading to suicide events” [18].

Natural language processing (NLP) and machine learning (ML) have the potential to complement clinical practice by categorizing and analyzing data from clinical notes [19]. NLP is a computerized process that analyzes and codes human language into text that [20] ML algorithms can analyze and use to predict outcomes [21]. ML approaches have been used to predict suicidality in research using clinical notes (accuracy > 65%) [22], patient text messages (sensitivity = 0.56) [23], healthcare administrative data (AUC = 0.84) [24], and from structured data found in adolescent’s EHR [25]. Research applying NLP and machine learning to classification of suicidal behavior among psychiatrically hospitalized adolescents is limited but needed because such youth have high clinical severity, a greater frequency of past self-harm, and a higher propensity for future self-harm [3, 26, 27].

Using NLP to codify pre-admission electronic health record (EHR) notes to detect suicidal behavior is a promising approach [28]. EHR clinician notes are likely to capture important correlates of suicidal behavior in aggregate over time since mental health clinicians are trained in biopsychosocial mental health evaluation, including risk assessment [29]. Further, diagnostic codes for suicidal behavior are proving inadequate for identifying research cohorts of youth with suicidal behavior. A study using NLP to examine EHR notes of suicidal patients found that only a small proportion of patients with suicidal ideation or attempt documented in EHR notes had a corresponding diagnostic code recorded in the EHR [30]. A study of adolescents with autism spectrum disorder developed an NLP classification tool with a recall of 0.91 for detection of suicidality in EHR notes [31]. However, such studies may fail to capture suicide attempts that are not overtly mentioned in the medical record. This approach has yet to be applied to studying suicide among psychiatrically hospitalized adolescents and may prove valuable, as adolescents have been found to be more likely to report suicide attempts under conditions of anonymity [32].

An algorithm based on unstructured notes may capture a broader spectrum of patients than one using structured diagnostic codes, which may not include all patients with a history of suicide attempt. EHR notes are also relatively frequent in this population (e.g. weekly psychotherapy and monthly medication visits, in addition to primary care, emergency, and inpatient treatment, and documentation of collateral contacts), which is important for detecting low prevalence outcomes like suicide attempt. Finally, NLP of clinical notes can allow for detection of novel variables that are specific to the particular health system under study.

To address this gap in the research literature, we describe the development of a machine learning algorithm that generates classification models from codes developed by NLP analysis of EHR notes in order to categorize adolescents by history of suicide attempt. We describe in detail the process and method used to capture codes from the clinical notes and the development and refinement of the machine learning algorithm. The algorithm yielded a modest classification capability, and a method for developing and refining similar algorithms in other psychiatric settings.

## Methods

### Sample

The IRB of the Cambridge Health Alliance approved this study (CHA-IRB-0886/01/12). Adolescents gave in person assent to participate in the survey and their legal guardian provided in person or telephone (audio-recorded) consent to participate and for analysis of electronic health record data. Participants aged 12 to 20 years old were recruited from an inpatient psychiatric unit of a community hospital in Massachusetts from February 2012 to September 2016. They were invited to complete a confidential self-report survey that assessed mental health and risk-taking behaviors, including history of suicidal thoughts and attempts. This was a research survey and neither clinicians nor parents were privy to the patients’ responses. Parents or guardians provided consent either in-person or by phone (audio recorded), and the adolescent patients provided in-person assent. Human subjects approval to survey patients and to analyze notes from their electronic health records was obtained from the institutional review board of the health system. The survey sample included a total of 241 respondents.

### Sample dataset

Of the total survey sample, 73 youth had at least one EHR documentation available for treatment visits in the year prior to the index psychiatric admission in the same health system. Patients in this sample are described in Table 1. EHR documentation included outpatient, inpatient, or emergency room clinical encounters across mental health and primary care. A total of 9415 notes were identified, ranging from 1 to 876 notes per patient, with a mean of 129 and median of 70 notes prior to admission. These clinical notes generally included clinician progress notes and clinician documentation of contacts with family members, other members of the treatment team, and relevant systems (e.g. schools, community service agencies). Notes were written by a variety of providers, including physicians, psychologists, nurses, case managers, social workers, and medical assistants.

**Table 1:** Sociodemographic and clinical descriptors of adolescent sample.

	No attempt	At least one attempt	P
	*n (%)*	*n (%)*	
**Suicide attempt in the past year**	46 (63.0%)	27 (37.0%)	
**Age in years**	*Mean (S*.*D*.*)*	*Mean (S*.*D*.*)*	>.10
	15.76 (1.55)	16.11 (1.76)	
**Insurance**	*n (%)*	*n (%)*	
Private	14 (30.4%)	10 (37.0%)	
Public	32 (69.6%)	17 (63.0%)	
**Sex**	*n (%)*	*n (%)*	
Male	20 (43.4%)	8 (29.6%)	
Female	26 (56.5%)	19 (70.3%)	
**Race/Ethnicity**	*n (%)*	*n (%)*	
Asian	2 (4.3%)	1 (3.7%)	
Black	7 (15.2%)	3 (11.1%)	
Hispanic	6 (13.0%)	10 (37.0%)	
White	31 (67.4%)	13 (48.1%)	
**Any use of clinical services in year prior to admission**	*%*	*%*	
Behavioral Health Inpatient	33%	33%	
Behavioral Health Outpatient	43%	56%	
Emergency Department	67%	67%	
Inpatient	2%	11%	
Outpatient	17%	30%	
Primary Care	43%	52%	

Categories for chi-squared test of race/ethnicity were dichotomized to “white” and “non-white” due to small cell sizes. S.D.: standard deviation

### Outcome variable of interest

The outcome variable of interest was any past year suicide attempt captured by an item from the Youth Risk Behavior Survey: “During the past 12 months, how many times did you actually attempt suicide?” The research literature supports the use of a self-report suicide history variable in inpatient settings. Adolescents are much more likely to self-report suicide attempt under conditions of anonymity [32], which our survey was. A survey of outpatient and inpatient adolescents in the UK showed that 20% reported at least one episode of self-harm on the questionnaire that was not recorded in the clinical record [33]. A study among adults showed 83% agreement between self-report of self-harm and therapist notes and, further, all medically treated episodes were reported by participants [34]. On the other hand, a study of adolescents found under-reporting of “self-harm” on a questionnaire when compared to hospital admission data [35]. Based on this evidence, we determined that self-report was an adequate gold standard variable to use for this study, and further chart review to augment self-report was not conducted.

Responses were dichotomized as zero or at least once. A total of 27 (37%) survey respondents reported at least one suicide attempt. Differences in demographic and clinical characteristics between participants with attempt and no attempt were compared using chi-square and t tests.

### Natural language processing of electronic health records

A dataset was created of all clinical notes for survey participants with EHR documentation for one year prior to the index admission (where the survey was completed). The NLP analysis used Invenio software [36] to codify the unstructured text of EHR records during that time period. Invenio is based on the open source Apache cTAKES system [37] and analyzes the unstructured free text of clinical notes to generate Concept Unique Identifiers (CUIs), which are alphanumeric codes representing specific items in the Unified Medical Language System (UMLS) [38]. For example, the CUI “C0424000” represents “Feeling suicidal (finding)”. Similar to the cTAKES platform, Invenio uses features such as a sentence boundary detector, tokenizer, normalizer, part-of-speech tagger, shallow parser, and named entity recognition annotator to convert free text into UMLS CUIs. Invenio also captures negation in the context of EHR sentences, such as “no suicide attempts”. A total of 11806 CUIs, including their negations, were extracted from the clinical notes of the total sample and used as data for the machine learning algorithm. This list of CUIs served as the library of eligible CUIs for the five-fold cross validation conducted for the algorithm development, described below.

To enrich the classification power of the CUIs identified through NLP, a “curated” list of 34 suicide-related predictive factors and 30 protective factors was developed by behavioral health clinicians on the research team, drawing from the literature on risk factors for adolescent suicide [39–42] (S1 Table). This curated list was transformed into CUIs by matching the curated terms to text-strings from EHR notes of the total sample.

### Machine learning and algorithm development

We used a random forest algorithm, an ensemble classification method with validated applications in mental health research [14, 25, 43], to classify each patient by history of past-year suicide attempt [44]. Random forest sits in the middle of the so-called “axes of machine learning,” relying on human selection and curation of variables that are analyzed using hundreds of decision trees to identify nonlinear variable interactions [45]. The analysis was performed in the statistical software R (version 3.4.4) using the package randomForest [46]. Random forest classification develops decision trees by creating nodes that are related or not to suicidality. For example, the word “depression” (main node) was split between presence or absence of whether the word depression appeared in the note [47].

We then performed five-fold cross-validation of the training set [48] in order to optimize the features (e.g., the set of CUIs) used in the final model. To do so, we first randomly partitioned the data into a training set (80% of patients, n = 58) and an out-of-sample validation set (20% of patients, n = 15). The first step in the cross-validation process was to randomly divide the training set into 5 mutually exclusive datasets, or folds. Two folds contained data from 11 patients while the remaining three folds contained data from 12 patients. During cross-validation, each fold served as the testing dataset for the model that was trained on the remaining four datasets. Thus, each patient in the 80% training set was assigned to a test dataset once (a probability of 20%), and to a training dataset four times.

Second, CUIs were extracted from the clinical notes of the patients from the full dataset. Invenio wrote each CUI contained in each note to a log, which was read by SAS version 9.4 (SAS Institute Inc. Cary, NC, USA), and a dummy variable was generated for each CUI. A matrix of 11806 dummy variables (CUIs) by 9415 notes (records) was generated. To reduce overfitting, we required that a CUI appear at least once for four different patients before including it in the random forests algorithm, which trimmed the matrix to approximately 4000 dummy variables. For the cross-validation and the model performance evaluation (conducted on the 20% out-of-sample dataset), models were trained only on CUIs that originated from notes in each fold’s training dataset.

Third, the 50 CUIs with the largest mean decrease in the Gini impurity index for each fold (S2 Table) were identified using random forest and combined with CUIs from the “curated” list that appeared in the training data for that particular fold. The Gini impurity index is a decision tree split quality measure used in machine learning corresponding to the mean decrease in impurity caused by a node [49]. It is one method of feature importance available in the random forest approach. The average number of CUIs used in this iterative step was 160.

Fourth, a model was built by generating 300 decision trees using the random forest procedure. Fifth, the resulting model was applied to each note of each individual randomized to the test fold to assess performance statistics for that fold. Finally, if the number of notes classifying suicide for an individual person was greater than a specified cutoff (e.g. 0%, 10%, 20%, 30%, 40%, 50%, 60%, 70%, 80%, 90%, or 100%), then the individual was classified as having attempted suicide in the past year. This process was repeated 5 times, with each subset of the training dataset (that is, 16% of the total sample) serving as a holdout dataset once. Statistics across the five folds were then averaged to select the cut-off (the proportion of notes indicating suicide attempt) that determined the best model.

### Evaluation of algorithm performance

A final model was built on the training dataset (80% of patients, n = 58), using the features from the best model determined by five-fold cross-validation (in this case, a model that used a 20% cutoff for number of notes classified as suicide attempt). The validity of the model was evaluated by performing validation on the out-of-sample dataset (20% of the total sample, n = 15 patients), determining the sensitivity, specificity, positive predictive value (PPV), negative predictive value (NPV), and accuracy in the comparison of individuals classified as suicide attempt by the algorithm and those who reported a suicide attempt in the gold standard survey measure obtained during the inpatient admission. We also assessed the receiver operating curve (ROC) and estimated the area under the curve (AUC) [14] for classifications that varied by the percentage of notes (0%, 10%, 20%, 30%, 40%, 50%, 60%, 70%, 80%, 90%, 100%) indicating suicide attempt.

## Results

A total of 27 patients reported at least one suicide attempt in the year prior to admission. This group was predominantly female, white or Hispanic, and had public insurance (Table 1). Comparisons of demographic and clinical information showed no significant differences between those with and without self-reported suicide attempt (p>0.10).

The random forest procedure identified EHR phrases that, when converted to CUIs in the UMLS, were significantly associated with suicide attempt. Examples of these EHR phrases are found in the S2 Table, which lists the top fifty phrases identified in each of the five training folds. There was a notable preponderance of EHR phrases representing known adolescent suicide risk factors. These included phrases related to suicidal behaviors as previously described in the literature, such as risk factors for suicidality (“suicide attempts,” “thoughts of suicide,” “mood; Depressed”; “pain”), family [50–52] (e.g. “fathers,” “brothers,” “grandfather,” “parents,” “siblings”), medication (antidepressants, antipsychotics, anti-inflammatories), and mental health conditions (“severe depression”, “PTSD,” “unspecified psychosis,” disorder, recurrent bipolar,” substance use/abuse,” “attention deficit hyperactivity disorder,”).

Regarding classification statistics for the machine learning model (Table 2), the optimal model we selected in prioritizing sensitivity without overly sacrificing specificity was a model that used a 20% cutoff for number of notes classified as suicide attempt. The performance across all cutoffs and folds of cross-validation can be viewed in S3 Table. The mean model performance at this cutoff included a sensitivity of 0.72 (range 0.25–1.00), a specificity of 0.26 (0.13–0.50), a PPV of 0.33 (0.13–0.75), an NPV of 0.63 (0.25–1.00). At a cutoff of 20%, the system had an accuracy of 42% (range 0.17–0.58), as compared to the most frequent class baseline of 63%.

**Table 2:** Mean model performance by cutoff (0–100%) Across 5 fold cross-validation.

Cutoff (%)	Sensitivity (Min—Max)	Specificity (Min—Max)	PPV (Min—Max)	NPV (Min—Max)	Accuracy (Min—Max)
0	1.00 (1.00–1.00)	0.00 (0.00–0.00)	0.36 (0.09–0.75)	0.00 (0.00–0.00)	0.36 (0.09–0.75)
10	0.87 (0.67–1.00)	0.15 (0.00–0.33)	0.36 (0.11–0.75)	0.55 (0.00–1.00)	0.38 (0.25–0.58)
20	0.72 (0.25–1.00)	0.26 (0.13–0.50)	0.33 (0.13–0.75)	0.63 (0.25–1.00)	0.42 (0.17–0.58)
30	0.51 (0.22–1.00)	0.50 (0.25–1.00)	0.38 (0.14–1.00)	0.61 (0.30–1.00)	0.42 (0.25–0.55)
40	0.42 (0.00–1.00)	0.74 (0.50–1.00)	0.26 (0.00–0.50)	0.66 (0.25–1.00)	0.54 (0.25–0.73)
50	0.25 (0.00–1.00)	0.88 (0.75–1.00)	0.10 (0.00–0.50)	0.65 (0.25–1.00)	0.59 (0.25–0.82)
60	0.00 (0.00–0.00)	0.88 (0.75–1.00)	0.00 (0.00–0.00)	0.62 (0.25–0.89)	0.55 (0.25–0.75)
70	0.00 (0.00–0.00)	0.95 (0.75–1.00)	0.00 (0.00–0.00)	0.63 (0.25–0.91)	0.61 (0.25–0.91)
80	0.00 (0.00–0.00)	0.98 (0.88–1.00)	0.00 (0.00–0.00)	0.63 (0.56–0.91)	0.63 (0.56–0.91)
90	0.00 (0.00–0.00)	1.00 (1.00–1.00)	0.00 (0.00–0.00)	0.64 (0.56–0.91)	0.64 (0.56–0.91)
100	0.00 (0.00–0.00)	1.00 (1.00–1.00)	0.00 (0.00–0.00)	0.64 (0.56–0.91)	0.64 (0.56–0.91)

Cutoff, percentage of notes in a patient’s record determined to be predictive of suicide attempt; PPV, positive predictive value; NPV, negative predictive value

**Fig 1:**
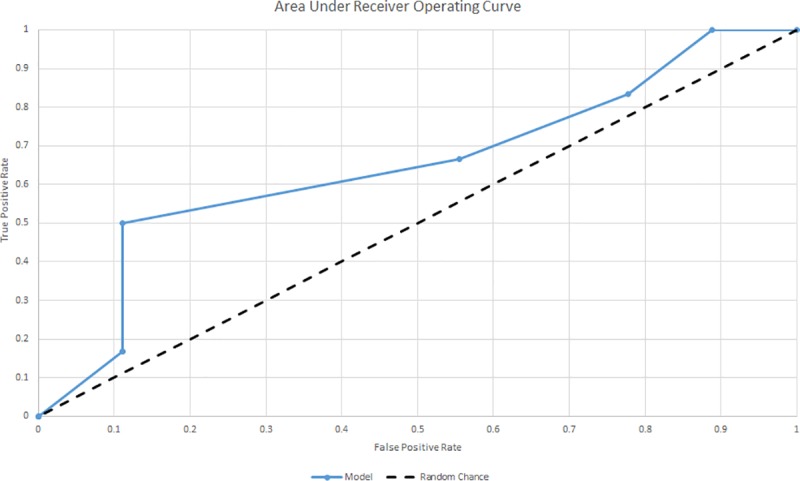
Area under receiver operating curve.

Using the model trained off the 80% dataset, and applying the 20% cutoff identified as optimal in the cross validation, we then performed external validation on the 20% out-of-sample dataset, reporting the same performance metrics. The final model had a sensitivity of 0.83, a specificity of 0.22, a PPV of 0.42, an NPV of 0.67, an accuracy of 0.47%, and an AUC of 0.68 (Fig 1). In sensitivity analyses, model performance was measured after decreasing or increasing the percentage of notes indicating suicide attempt. These performance metrics are presented in Table 3, with each row representing a different “cutoff” for the minimum proportion of EHR notes required to indicate suicide attempt.

**Table 3:** Model performance by cutoff (0–100%).

			Actual attempt						
Cutoff			No	Yes	Sensitivity	Specificity	PPV	NPV	Accuracy
0%	**Classified attempt**	**No**	0	0	1.00	0.00	0.40	0.00	0.40
		**Yes**	9	6					
10%	**Classified attempt**	**No**	1	0	1.00	0.11	0.43	1.00	0.47
		**Yes**	8	6					
20%	**Classified attempt**	**No**	2	1	0.83	0.22	0.42	0.67	0.47
		**Yes**	7	5					
30%	**Classified attempt**	**No**	4	2	0.67	0.44	0.44	0.67	0.53
		**Yes**	5	4					
40%	**Classified attempt**	**No**	8	3	0.50	0.89	0.75	0.73	0.73
		**Yes**	1	3					
50%	**Classified attempt**	**No**	8	5	0.17	0.89	0.50	0.62	0.60
		**Yes**	1	1					
60%	**Classified attempt**	**No**	8	5	0.17	0.89	0.00	0.62	0.60
		**Yes**	1	1					
70%	**Classified attempt**	**No**	9	6	0.00	1.00	0.00	0.60	0.60
		**Yes**	0	0					
80%	**Classified attempt**	**No**	9	6	0.00	1.00	0.00	0.60	0.60
		**Yes**	0	0					
90%	**Classified attempt**	**No**	9	6	0.00	1.00	0.00	0.60	0.60
		**Yes**	0	0					
100%	**Classified attempt**	**No**	9	6	0.00	1.00	0.00	0.60	0.60
		**Yes**	0	0					

## Discussion

In this proof of concept study, we present a method for developing a classification model for past-year suicide attempt among psychiatrically hospitalized adolescents using natural language processing and machine learning of clinical narratives from electronic health record data preceding admission. From a sample of 73 patients admitted to a psychiatric unit, we developed a classification algorithm with moderate sensitivity and negative predictive value, a modest AUC, and an accuracy below the most frequent class baseline. To the best of our knowledge, this is the first time unstructured data has been analyzed using NLP to develop a machine learning classification algorithm in an adolescent inpatient population. This initial signal of success using a small sample should be validated in larger datasets. The approach demonstrates how EHR notes, enriched with clinically relevant information for adolescent suicide attempt, can be used to identify youth with histories of suicidal behavior, which can aid inpatient treatment planning during a particularly vulnerable time for this high risk population.

Machine learning-based algorithms can lead to methodologically sound but clinically inadequate results due to the lack of clinical context included in the model [53, 54]. This limitation may reveal the benefit of including data derived from natural language processing of clinical notes. EHR notes, especially those from mental health settings, contain a richness of description and context that, when organized for higher level analysis, may identify important relationships with adverse health care outcomes. The model we describe in this paper is therefore notable for its use of NLP-derived phrases for use in the random forest procedure, as opposed to only structured data (e.g. from drop-down and forced selection EHR fields). For example, the algorithm described in this paper yielded a high number of EHR phrases associated with family, which may demonstrate the importance of familial support and/or conflict in understanding suicidal behavior among adolescents with severe mental health difficulties. This relationship between family and suicide attempt is supported by prior machine learning research [50] as well as studies using other research methods [51, 52, 55–57]. Future studies can then explore the direction of these relationships to guide clinicians towards the most relevant family-related history to assess, document, and address in treatment planning. Thus the value in using NLP to a health care institution may be that the resulting classification model is attuned to the practice behaviors and patient characteristics of that particular setting.

It is important to note that the performance of the algorithm reported here varied by the threshold of notes that were indicating suicide attempt. Optimizing for sensitivity, we selected a cutoff of 20% of positive notes. To our knowledge, this is the first paper to report these cutoffs and how the classification statistics vary on this parameter. Users of such algorithms can let the clinical need of the tool guide the optimal cut-off in their setting and the related trade-offs between sensitivity and specificity.

Comparing the test metrics of our algorithm to prior research, we note first that there is no common practice of presenting algorithm performance in the literature, although useful guidelines have been published to help standardize the field [58]. Some report performance statistics and probabilities, while others present area under the curve [14]. A study using a self-report measure to determine suicide attempt history achieved sensitivities ranging from 55.8–72.1% and AUC’s ranging from 0.65–0.77 [59]. This study did not use EHR data, but rather measures of sociodemographic and clinical variables (e.g. a depression rating scale). Thus, although the studies are similar, the differences in methodology make it difficult to compare the capabilities of the models.

In the prediction literature, a recent study that also used random forest took care to stratify predictions across a two year period, showing increasingly accurate predictions more proximal to the index attempt (AUCs > 0.83) [25]. Similar to our NLP findings, this paper also found psychotropic medications to be important predictors of suicide attempt.

One potential clinical application of such an algorithm would be to serve as an alert in an EHR system to complement existing risk scales and to aid in clinical risk assessment. Such alerts have been shown to improve recognition of other chronic health conditions, such as pediatric hypertension, although recognition differed by race/ethnicity and gender [60]. A small trial among suicidal adolescents showed a significant increase in safety planning, but with “moderate” satisfaction reported by clinicians [60]. Further research is needed to safely incorporate the signals of automated algorithms into the regular workflows of inpatient treatment planning, in a way that is meaningful to clinicians and families [61].

### Limitations

The suicide attempt outcome used in this study allows for the possibility that the algorithm is in fact predicting a suicide attempt. For example, in the event that an individual in the dataset was hospitalized immediately following a suicide attempt, the algorithm using notes in the year prior to that attempt and subsequent hospitalization would, in this case, be predicting an attempt in the future. The specific dates of suicide attempt are unavailable in the survey and so we are unable to determine whether the algorithm is classifying existing events or predicting future events. In future research, the algorithm could be easily re-oriented completely towards prediction by entering only data from EHR notes that come definitively before a suicide outcome with known dates (e.g. an ICD10 code for a suicide attempt documented in an emergency department visit).

Our approach was limited by a small dataset from one community health system, which limits generalizability of findings. The use of natural language processing to define the variables eligible for the random forest procedure limits generalizability further, since they reflect the particular patient, clinician, and treatment settings characteristics within the community health system [62]. Analysis of free text in clinical notes presents both challenges and opportunities due to differences in provider documentation. In comparison, the use of standardized terminology in radiology and cancer staging have resulted in better model performance [63].

The accuracy of classification models using NLP may be improved by including complementary structured variables in the dataset (such as diagnoses, medications, outcome measures, and social determinants) to augment the detection of suicide attempt [25, 64]. For example, suicide outcomes from the electronic record, such as ICD-10 codes for suicide and suicide attempt, provide valid evidence of treatment for self-harm, are less prone to patient response bias, and are therefore suitable for future research in this area. However, such codes would not capture attempts that went untreated, and prior research shows that such codes are under-utilized by clinicians [30], perhaps because they do not indicate a billable diagnosis (e.g. Major Depressive Disorder). By using a self-report variable as the gold-standard outcome, we avoided the under-reporting bias known to affect ICD-9 coding of self-injury [65]. The use of a brief screener for suicide attempt with hospitalized adolescents may also have been vulnerable to a higher false positive rate than longer, structured measures [66]. We look forward to validating this work using such outcomes, in larger samples, and across multiple treatment settings. A validated prediction algorithm can then be studied clinically to determine safe implementation practices from the perspectives of clinicians and the families they serve.

## Supporting information

S1 Table List of “curated” concepts related to adolescent suicide attempt. (XLSX)

S2 Table Top fifty concept unique identifiers from each validation fold. CUI, Concept Unique Identifiers; UMLS, Unified Medical Language System; Gini impurity index, a measure used in machine learning corresponding to the mean decrease in impurity caused by a node; EHR: electronic health records. UMLS CUI: alphanumerical code designated by UMLS to name each CUI. Concept: Description of CUI, obtained from 2018AA release of the UMLS Metathesaurus Browser. EHR text phrase: text phrase found in electronic health record matching the CUIs.
(XLSX)

S3 Table Model performance by cutoff and fold. (XLSX)

S1 File Example data. Zipped folder containing SAS data files that were created by the research team. These files were not constructed from patient data and are intended for demonstration of our methods. Files include CUI logs, 5 folds of example training data, a file containing data from a sample of “patients” who self-reported past year suicide attempt, a file containing data from a sample of “patients” who did not report a past year suicide attempt, the SAS code used for the analysis, and a modified version of %PROC_R (Wei, X. (2012). %PROC_R: A SAS Macro that Enables Native R Programming in the Base SAS Environment. Journal of Statistical Software, 46(Code Snippet 2), 1–13.), that calls R from SAS.
(ZIP)
